# The course of health status and (health-related) quality of life following fracture of the lower extremity: a 6-month follow-up study

**DOI:** 10.1007/s11136-015-1167-4

**Published:** 2015-11-05

**Authors:** M. A. C. Van Son, J. De Vries, J. A. Roukema, T. Gosens, M. H. J. Verhofstad, B. L. Den Oudsten

**Affiliations:** CoRPS, Department of Medical and Clinical Psychology, Tilburg University, P.O. Box 90153, 5000 LE Tilburg, The Netherlands; Department of Surgery, St. Elisabeth Hospital, Tilburg, The Netherlands; Department of Medical Psychology, St. Elisabeth Hospital, Tilburg, The Netherlands; Department of Orthopaedics, St. Elisabeth Hospital, Tilburg, The Netherlands; Trauma Research Unit, Department of Surgery, Erasmus MC, University Medical Center Rotterdam, Rotterdam, The Netherlands

**Keywords:** Health status, Health-related quality of life, Quality of life, Lower extremity fractures

## Abstract

**Purpose:**

The aim of this prospective study was to describe the course of health status (HS), health-related quality of life, and quality of life (QOL) in patients with lower extremity fractures (LEF) up to 6 months post-fracture.

**Methods:**

Patients (*n* = 171; age range 18–100 years) completed the World Health Organization Quality of Life assessment instrument-Bref (WHOQOL-Bref) and the Short Musculoskeletal Function Assessment questionnaire (SMFA) at time of diagnosis (i.e., pre-injury status), 1 week, and 6 months post-fracture. Linear mixed modeling was performed.

**Results:**

Interaction effects of time with treatment were detected for the WHOQOL-Bref facet Overall QOL and General health (*p* = .002) and Physical health (*p* = .003). Patients did not return to their pre-injury Physical health, Psychological health, and Environment 6 months post-fracture (*p* < .05). No effects were found for Social relationships. The SMFA subscale Lower extremity dysfunction showed main effects for time and treatment (*p* < .0001) with full recovery at 6 months (*p* = .998). An interaction effect of time with treatment was found for Daily life consequences (*p* < .0001) with nonoperatively treated patients showing full recovery (*p* = 1.00), whereas surgically treated patients did not (*p* = .002).

**Conclusions:**

Six months after LEF, patients still experienced impaired physical and psychological health on the WHOQOL-Bref compared to their pre-injury status. However, patients showed full recovery on SMFA Lower extremity dysfunction, indicating that the choice of the questionnaire influences the derived conclusions. LEF did not affect satisfaction with social relationships.

## Introduction

Fractures of the proximal femur, ankle, and metatarsal encompass 27 % of the fractures that orthopedic surgeons encounter in their daily practice (i.e., incidence 129.4, 100.8, and 75.4 fractures per 10^5^ person-years, respectively) [1]. After a lower extremity fracture (LEF), physical impairment often sustains 6 months or longer [2–5]. For instance, 6 months after LEF, more than half of the patients still report deficits in range of motion, and in 12–25 % of the patients, loss of muscle strength was present [2]. About half of the patients with a surgically treated bimalleolar or trimalleolar fracture has residual pain, stiffness, and ankle swelling 1 year post-fracture [3]. Even 6.5 years after surgical treatment of a patella fracture, patients still experience extensor muscle deficits [4]. In conclusion, LEF can contribute to short- and long-term impairment of the patient. Almost one-third of the patients with a hip fracture do not return to their pre-injury fracture levels of daily functioning within 1 year post-fracture [5].

Research indicates that physical functioning and the activity level of patients with fractures cannot be based only on X-ray indices [6]. Moreover, in patients with LEF, the relationship between lower extremity impairment (i.e., range of motion, muscle strength, and pain) and the ability to perform daily life activities (e.g., home management, work, and recreation) was considered small [7, 8]. Therefore, the patients’ perspective on the recovery process after LEF, reflected by their health status (HS), health-related quality of life (HRQOL), and quality of life (QOL), will offer valuable information in addition to radiographic and absolute physical outcomes. HS, HRQOL, and QOL have in common that at least the physical, psychological, and social domains are included. However, HS is merely a self-reported assessment of functioning [9], whereas QOL also encompasses patients’ subjective evaluations of their functioning and well-being [10–12]. HRQOL concerns those QOL components that are impacted by the disease [13].

However, HS, HRQOL, and QOL are not well examined in trauma patients with fractures of the extremities [14, 15]. More specifically, to our knowledge, only seven studies on LEF applied a HS instrument, the Short Form Health Survey-36 (SF-36) [16], and the Sickness Impact Profile (SIP) [17], and none of the studies used HRQOL or QOL instruments in these patients [2, 7, 8, 18–21]. Research within the field of oncology indicates that outcomes after a disability can be different depending on either the application of a HS or QOL instrument [22]. This might be explained by the double subjectivity of a QOL instrument, i.e., the perceived level of satisfaction is important in addition to the appraisal of level of functioning measured with HS [13]. For example, a patient with an ankle fracture may be limited in climbing the stairs (i.e., HS). However, it is possible that the patient is not bothered by this (i.e., QOL) because the patient lives in an apartment with an elevator.

Therefore, with the absence of HRQOL and QOL outcomes in LEF, our aim was to describe the course of HS, HRQOL, and QOL in patients up to 6 months after LEF using the generic World Health Organization Quality of Life assessment instrument-Bref (WHOQOL-Bref) [23] and the Short Musculoskeletal Function Assessment questionnaire (SMFA) [24]. We expected that patients’ HS, HRQOL, and QOL would be worse 1 week after fracture compared to their pre-injury status, but that patients would report improved HS, HRQOL, and QOL 6 months post-fracture. However, based on the existing literature [2–5], we also hypothesized that patients would not completely recover 6 months post-fracture, in particular those patients who were surgically treated (i.e., who were expected to have higher injury severity compared to patients nonoperatively treated).

## Materials and methods

### Patients

Patients were recruited during the period November 2010 until January 2012 at the St. Elisabeth Hospital, Tilburg. Main inclusion criteria were a unilateral LEF (i.e., fracture inflicted by trauma and confirmed by X-ray) and a minimal age of 18 years. Multiple trauma patients were excluded from the study as well as patients with the diagnosis of a pathological or open fracture. An incapacity to complete the self-report measures by the patients themselves, due to insufficient knowledge of the Dutch language or neurological conditions (e.g., dementia), was a reason for exclusion. Also the presence of severe psychopathology (i.e., suicidal, self-mutilation, history of psychoses) or serious physical comorbidity (e.g., heart failure, lung cancer) were exclusion criteria.

### Design

Before patients were invited to participate, their eligibility was checked based on the registered International Classification of Diseases-10 (ICD-10) code at the Emergency Department. Other relevant information referring to our inclusion and exclusion criteria was extracted from the electronic patient charts. Eligible patients were invited to participate in the study during their visit of the Emergency Department, or within a few days after this visit, by the medical staff of the Emergency Department or by a member of the research group. Patients were asked to complete a set of self-report measures at three time points. At time of fracture diagnosis, patients were asked to report on their condition of the past 2 weeks before fracture occurrence to collect information on their pre-injury HS, HROQL, and QOL (i.e., to establish a baseline: Time-0_retrospective_). One week post-fracture (Time-1) and 6 months post-fracture (Time-2), patients received the same paper questionnaire booklets by mail with the instruction to report about their present condition. If patients did not return the first questionnaire set they were still invited at Time-1 and Time-2 to participate by completing the booklets of these two later time points.

### Measures

Patients completed the WHOQOL-Bref [23] and the SMFA [24]. The WHOQOL-Bref is a generic QOL questionnaire of 26 items that measures four domains: Physical health, Psychological health, Social relationships, and Environment. Additionally, the questionnaire has a general evaluative facet, consisting of two items, that assesses patients’ overall QOL and general health [23]. The psychometric properties of the WHOQOL-Bref are demonstrated to be good in various patient populations ranging from psychiatric outpatients [25] to patients with severe joint disease [26] and women with breast cancer [27].

The Dutch adaptation of the SMFA [24] measures HS and HRQOL with 53 items in patients with musculoskeletal disorders and is a psychometrically sound measure in patients with fractures [24]. We reported on the subscales Lower extremity dysfunction and Daily life consequences (and choose not to report on the subscale Upper extremity dysfunction) because these subscales were regarded as most informative in LEF.

### Statistical analysis

Linear mixed models with a pre-specified covariance pattern model were performed to examine the course of HS, HRQOL, and QOL over time. In this type of analysis, a patient is included if information on at least one time point is available, thereby using the available information more efficiently compared to repeated-measures (M)ANOVA. In repeated-measures (M)ANOVA, patients are only included in the analyses if they complete all time points. This is often a problem because of missing data in longitudinal studies [28].

The predictors included in the model were the categorical variables time with three levels (i.e., Time-0_retrospective_, Time-1, Time-2) and type of treatment with two levels (i.e., nonoperative treatment versus surgical treatment). Treatment type was included as a proxy variable of injury severity, assuming that the nonoperatively treated group of patients has lower injury severity compared to the surgically treated group. Analyses were corrected for age. A statistical power analysis was performed, with the use of G*Power [29], which indicated a sample size needed of 45 patients for each group (i.e., nonoperatively treated group versus surgically treated group) to detect moderate differences between the three time points. The error covariance pattern was tested for each WHOQOL-Bref domain and SMFA subscale in a model with time as predictor and with restricted maximum likelihood. Either the unstructured error covariance pattern was applied if the log likelihood ratio test was significant, or the compound symmetry error covariance pattern was used if the log likelihood ratio test showed insignificant.

The possible interaction of time and type of treatment was examined. Estimated marginal means were used. If an interaction effect was found, simple effects were performed to determine whether there was a simple effect for time in the two separate treatment groups. Subsequently, if a simple effect of time was established for the treatment group, post hoc tests with Bonferroni correction were performed to determine which time points differed significantly. However, if no interaction effect was found, we subsequently removed the interaction term from the model and examined the main effects. If a main effect of time was found, post hoc tests for time with Bonferroni correction were performed. The course of HS and (HR)QOL for the nonoperatively and surgically treated groups was presented in line figures. Moreover, the WHOQOL-Bref norm scores of a healthy population are depicted in these figures [30, 31].

Cohen’s *d* was calculated as the model estimated mean difference divided by the model estimated pooled standard deviation of Time-0_retrospective_. In addition, the corresponding estimated confidence intervals of Cohens’ *d* were computed as the estimated lower and upper bounds of the 95 % confidence interval divided by the model estimated pooled standard deviation of Time-0_retrospective_. Effect sizes (i.e., Cohen’s *d*) between .20 and .50 were considered small, effect sizes of >.50 moderate, and >.80 large [32]. All statistical analyses were performed using IBM SPSS Statistics for Windows version 19 with a 5 percent level of significance.

## Results

A total of 171 patients returned at least one of the questionnaire booklets at Time-0_retrospective_, Time-1, or Time-2 (Table 1). Mean age was 49.7 years old (SD = 16.8), and 56.7 % of the patients was female (Table 2). In addition, most LEF were located at the ankle or foot (57.3 %). The majority of the patients were nonoperatively treated (59.6 %).

**Table 1:** Response patterns of the patients included in the analyses and number of data points used in the analyses

Response pattern for the three measurement points and number of data points				
	Time-0_retrospective_	Time-1	Time-2	Number of patients
	Pre-injury status	1 week post-fracture	6 months post-fracture	for each response pattern (total *N* = 171)
	**+**	**+**	**+**	66
	**+**	**+**	**−**	32
	**+**	**−**	**+**	11
	**−**	**+**	**+**	24
	**+**	**−**	**−**	16
	**−**	**+**	**−**	11
	**−**	**−**	**+**	11
Number of data points used in the analyses:	125	133	112	

‘+’ the patient has completed the questionnaire set on the given time point; ‘**−**’ the patient has not provided information on the time point by not returning the questionnaire booklet or by having too many missing items (i.e., more than 20 % of the items in a subscale)

Each patient can deliver a total of three data points (i.e., by completion of all three questionnaire sets). However, a patient might have only two data points by completing, for instance, only the first and third questionnaire sets. Therefore, it is possible to distinguish seven different response patterns. In mixed model analysis, a patient is included if at least one data point of this patient is available (i.e., not only complete cases). Therefore, this type of analysis is able to use the information of patients that missed one or two time points

**Table 2:** Patient’s characteristics at Time-0_retrospective_ for the total sample and stratified by treatment condition

	Total *N* = 171	Nonoperative treatment *N* = 102	Surgical treatment *N* = 69
Age (years)	49.7 ± 16.8 (52.0, 18–100)	48.3 ± 16.0 (52.0, 18–85)	51.7 ± 17.6 (52.0, 18–100)
18–64	142 (83.0)	89 (87.3)	53 (76.8)
65–74	22 (12.9)	10 (9.8)	12 (17.4)
75–84	3 (1.8)	2 (2.0)	1 (1.4)
85–94	3 (1.8)	1 (1.0)	2 (2.9)
≥95	1 (0.6)	0 (0.0)	1 (1.4)
Sex			
Male	74 (43.3)	42 (41.2)	32 (46.4)
Female	97 (56.7)	60 (58.8)	37 (53.6)
Marital status			
Partner	129 (75.4)	77 (75.5)	52 (75.4)
No partner	30 (17.5)	19 (18.6)	11 (15.9)
Missing	12 (7.0)	6 (5.9)	6 (8.7)
Educational level			
Low education: high school or less	58 (33.9)	38 (37.3)	20 (29.0)
High education: additional education after high school	96 (56.1)	54 (52.9)	42 (60.9)
Missing	17 (9.9)	10 (9.8)	7 (10.1)
Anatomical location of fracture			
Toe	25 (14.6)	25 (24.5)	0 (0.0)
Ankle/foot	98 (57.3)	69 (67.6)	29 (42.0)
Lower leg/knee	20 (11.7)	7 (6.9)	13 (18.8)
Upper leg/hip	28 (16.4)	1 (1.0)	27 (39.1)

A total of 171 patients returned at least one of the questionnaire sets. All values, except for the first row on age (mean ± SD with the median followed by the minimum and maximum between parentheses), are given as the number of patients, with the percentage in parentheses

There were no significant differences on age [*t*(169) = −1.283; *p* = .201], sex [*χ*^2^(1) = .453; *p* = .532], marital status [*χ*^2^(1) = 1.291; *p* = .310], and educational level [*χ*^2^(1) = .135; *p* = .837] between the group patients nonoperatively treated and surgically treated. However, as expected, the two groups differed significantly on fracture location [*χ*^2^(3) = 63.26; *p* < .0001]. Patients surgically treated had significantly more often fractures of the lower leg/knee (*p* = .027) or upper leg/hip (*p* < .0001) compared to patients nonoperatively treated. The nonoperatively treated group consisted of significantly more fractures of the toe (*p* < .0001) or ankle/foot (*p* = .001) than the group receiving surgical treatment.

### Course of QOL assessed with the WHOQOL-Bref

**Fig. 1:**
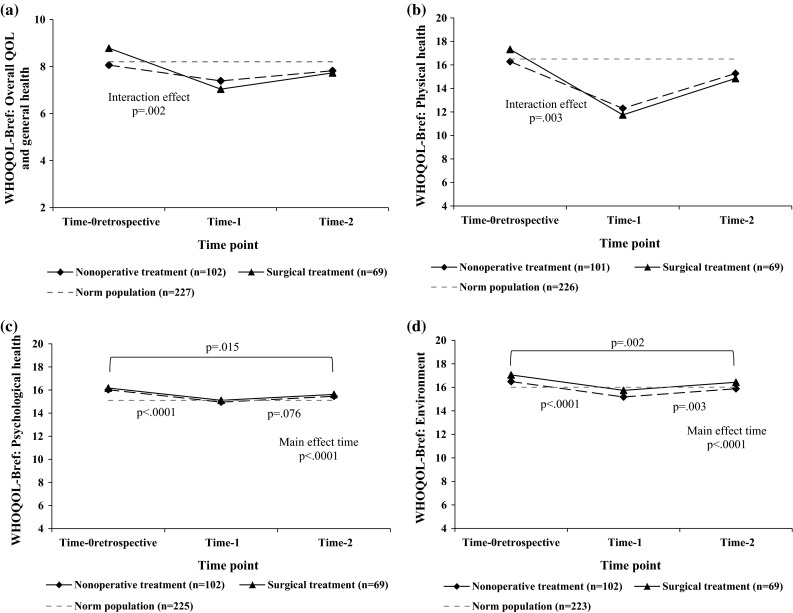
The course of **a** Overall QOL and General health, **b** Physical health, **c** Psychological health, and **d** Environment over time measured with the WHOQOL-Bref in patients with lower extremity fractures. *Note* Estimated marginal means are shown. A higher score indicates a better quality of life. An interaction effect for time with treatment was found (**a** *p* = .002; **b** *p* = .003). A main effect for time was found for the domain **c** Psychological health (*p* < .0001) and **d** Environment (*p* < .0001). The norm scores presented are derived from a healthy population. *Abbreviations* Time-0_retrospective_, pre-injury status; Time-1, 1 week post-fracture; Time-2, 6 months post-fracture; QOL, Quality of Life; WHOQOL-Bref, World Health Organization Quality of Life assessment instrument-Bref

For the facet Overall QOL and General health, an interaction effect of time with treatment (*F* = 6.360; *p* = .002) was demonstrated (Fig. 1a). Both in the nonoperatively and surgically treated groups, simple effects for time were found (*F* = 8.80; *p* < .0001 and *F* = 21.22; *p* < .0001, respectively). In nonoperatively treated patients, Overall QOL and General health deteriorated from Time-0_retrospective_ to Time-1 (*p* < .0001). The improvement in scores from Time-1 to Time-2 was significant (*p* = .042), and patient returned to their pre-injury levels (*p* = .351). In the surgically treated group, scores significantly declined from Time-0_retrospective_ to Time-1 (*p* < .0001) and did not significantly improve from Time-1 to Time-2 (*p* = .066). Six months post-fracture, patients did not return to their pre-injury levels (Time-0_retrospective_ and Time-2; *p* < .0001).

An interaction effect of time with treatment (*F* = 6.04; *p* = .003) was found on Physical health (Fig. 1b). In both treatment groups, simple effects for time were found (*F* = 74.61; *p* < .0001 and *F* = 62.78; *p* < .0001, respectively, for the nonoperatively treated group and surgically treated group). The scores significantly declined from Time-0_retrospective_ to Time-1 (*p* < .0001). Although a significant improvement in Physical health scores was found from Time-1 to Time-2 (*p* < .0001), patients did not return to their pre-injury status (Time-0_retrospective_ and Time-2; *p* = .001 for the nonoperatively treated group, *p* < .0001 for the surgically treated group) disregarding type of treatment.

For the domain Psychological health, no interaction effect (*F* = 2.33; *p* = .101) or main effect of treatment was found (*F* = .05; *p* = .820), but a main effect of time was detected (*F* = 22.68; *p* < .0001). Scores significantly declined from Time-0_retrospective_ to Time-1 (*p* < .0001). Patients did not recover from Time-1 to Time-2 (*p* = .076) and had still a significantly worse Psychological health 6 months post-fracture compared to their pre-injury levels (Time-0_retrospective_ and Time-2; *p* = .015) (Fig. 1c).

Regarding the domain Social relationships, no interaction effect (*F* = .06; *p* = .945) or main effects of either time (*F* = 2.04; *p* = .132) or treatment (*F* = .95; *p* = .332) were found.

The domain Environment (Fig. 1d) showed no interaction effect (*F* = .64; *p* = .530) or main effect of treatment (*F* = 1.69; *p* = .196). However, a main effect of time was indicated (*F* = 37.84; *p* < .0001). A significant deterioration of scores was found from Time-0_retrospective_ to Time-1 (*p* < .0001). These scores significantly improved from Time-1 to Time-2 (*p* = .003), but scores were still significantly worse 6 months post-fracture compared to pre-injury status (*p* = .002). Effect sizes (Cohen’s *d*) for the WHOQOL-Bref are shown in Table 3 with large effect sizes for the facet Overall QOL and General health and the domain Physical health. Moderate effect sizes were found for Psychological health and Environment.

**Table 3:** Effect sizes (Cohen’s *d*) for the WHOQOL-Bref and SMFA comparing Time-0_retrospective_ with Time-1 and Time-0_retrospective_ with Time-2

	Effect size (Cohen’s *d*)			
	Time-0_retrospective_ and Time-1		Time-0_retrospective_ and Time-2	
	Nonoperatively treated	Surgically treated	Nonoperatively treated	Surgically treated
WHOQOL-Bref				
Physical health	−1.51 (−1.78, −1.24)	−2.12 (−2.48, −1.75)	−0.38 (−0.62, −0.14)	−0.94 (−1.23, −0.64)
Psychological health	−0.45^a^ (−0.59, −0.30)	−0.45^a^ (−0.59, −0.30)	−0.23^a^ (− 0.39, −0.07)	−0.23^a^ (−0.39, −0.07)
Social relationships	^b^	^b^	^b^	^b^
Environment	−0.53^a^ (−0.66, −0.41)	−0.53^a^ (−0.66, −0.41)	−0.25^a^ (−0.39, −0.11)	−0.25^a^ (−0.39, −0.11)
Overall QOL and General health	−0.43 (−0.65, −0.20)	−1.10 (−1.41, −0.79)	−0.15 (−0.40, 0.09)	−0.67 (−0.98, −0.35)
SMFA				
Lower extremity dysfunction	1.95^a^ (1.71, 2.19)	1.95^a^ (1.71, 2.19)	0.10^a^ (−0.10, 0.30)	0.10^a^ (−0.10, 0.30)
Daily life consequences	0.82 (0.60, 1.05)	1.25 (0.95, 1.56)	−0.03 (−0.23, 0.17)	0.62 (0.37, 0.87)

WHOQOL-Bref, World Health Organization Quality of Life assessment instrument-Bref; SMFA, Short Musculoskeletal Function Assessment questionnaire; QOL, Quality of life; Time-0_retrospective_, pre-injury status; Time-1, 1 week post-fracture; Time-2, 6 months post-fracture

^a^Effect sizes are the same for the nonoperatively treated and surgically treated patients because no interaction effect was found

^b^Effect sizes are not presented because no statistically significant effect for time or interaction effect of time with treatment was found for this domain or subscale. The estimated confidence intervals of Cohen’s *d* are presented between square brackets

### Course of HS and HRQOL assessed with the SMFA

**Fig. 2:**
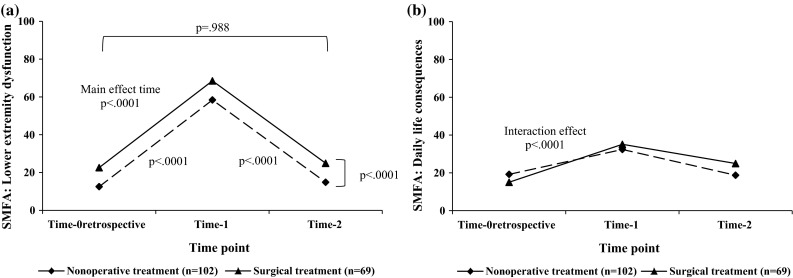
The course of **a** Lower extremity dysfunction, **b** Daily life consequences over time measured with the SMFA for patients nonoperatively treated and surgically treated. *Note* Estimated marginal means are shown. A higher score indicates worse health status or health-related quality of life. **a** A main effect for time (*p* < .0001) and treatment (*p* < .0001) was found. **b** An interaction effect for time with treatment was found (*p* < .0001). *Abbreviations* Time-0_retrospective_, pre-injury status; Time-1, 1 week post-fracture; Time-2, 6 months post-fracture; SMFA, Short Musculoskeletal Function Assessment questionnaire

No interaction effect was found for the subscale Lower extremity dysfunction (*F* = 2.615; *p* = .077). However, this subscale showed main effects of time (*F* = 178.40; *p* < .0001) and treatment (*F* = 20.12; *p* < .0001) (Fig. 2a). Patients significantly deteriorated on Lower extremity dysfunction from Time-0_retrospective_ to Time-1 (*p* < .0001). However, patients recovered from Time-1 to Time-2 (*p* < .0001) and returned to their pre-injury levels of lower extremity functioning 6 months post-fracture (Time-0_retrospective_ and Time-2; *p* = .998).

The subscale Daily life consequences showed an interaction effect of time with treatment (*F* = 8.15; *p* < .0001; Fig. 2b). Simple effects for time were shown in both treatment groups (i.e., nonoperatively treated *F* = 31.74; *p* < .0001 and surgically treated *F* = 29.39; *p* < .0001). In nonoperatively treated patients, the Daily life consequences scores significantly deteriorated from Time-0_retrospective_ to Time-1 (*p* < .0001). Six months post-fracture, patients had fully recovered (Time-0_retrospective_ and Time-2; *p* = 1.00) compared to their pre-injury scores by a significant improvement on the subscale Daily life consequences from Time-1 to Time-2 (*p* < .0001). A different pattern was detected for surgically treated patients. Although also a significant deterioration from Time-0_retrospective_ to Time-1 (*p* < .0001) was established and a significant improvement from Time-1 to Time-2 (*p* = .001), patients did not return to their pre-injury Daily life functioning (Time-0_retrospective_ and Time-2; *p* = .002). In Table 3, also the effect sizes (Cohen’s *d*) for the SMFA are shown with a large effect size for Lower extremity dysfunction comparing Time-0_retrospective_ and Time-1. However, comparing Time-0_retrospective_ and Time-2, effect sizes were below .20 on this subscale. Surgically treated patients showed moderate-to-large effect sizes on Daily life consequences.

## Discussion

The primary aim of this study was to describe the course of HS, HRQOL, and QOL of the whole range of patients with LEF with no restrictions applied during the inclusion of patients regarding age, patients admitted to the hospital, treatment type, or certain LEF fracture types found in prior research [2, 19–21]. We applied a generic QOL instrument (i.e., WHOQOL-Bref) and used a disease-specific instrument measuring HS and HRQOL (i.e., SMFA). Prior research on LEF applied only HS instruments [2, 7, 8, 18–21].

In summary, the WHOQOL-Bref domains showed an interaction effect of time with treatment or a main effect of time, except for Social relationships. Having a LEF had the greatest adverse impact on Physical health (i.e., compared to the other WHOQOL-Bref domains) with effect sizes (i.e., Cohen’s *d*) ranging from −0.38 to −2.12 up to 6 months after fracture. Prior research on patients that were diagnosed with a fracture (i.e., 54 % LEF) indicated that physical HS was significantly lower at a mean of 6 months post-fracture compared to US norms [18]. We found that patients did not fully recover on Physical health 6 months post-fracture, both in the surgical and nonoperative groups. On the SMFA subscale Daily life consequences, the surgically treated patients (i.e., higher injury severity) showed no full recovery 6 months post-fracture, whereas nonoperatively treated patients (i.e., lower injury severity) returned to their pre-injury levels.

For the whole group of patients, scores on Psychological health of the WHOQOL-Bref significantly deteriorated from pre-injury to 1 week post-fracture and patients had not recovered yet 6 months after their injury. Bhandari et al. [18] reported mental HS scores similar to US norms 6 months post-fracture. However, HS is a self-reported assessment of functioning without the subjective evaluation incorporated by QOL, thereby being different concepts and not directly comparable [9–12].

Results of the WHOQOL-Bref social relationships domain indicated that satisfaction with social relationships did not change over time despite having a LEF. However, our finding is in contrast with previous studies [2, 19] in which patients with LEF indicated 6 months post-fracture worse Social interaction category scores on the SIP [17] compared to pre-injury status. The conceptual differences of the used self-report measures (i.e., SIP versus WHOQOL-Bref) are a possible explanation. The SIP is a HS measure with questions such as: ‘I am going out less to visit people’ [17]. Patients with LEF may be restricted to their homes, but this does not mean significant others cannot visit the patient or cannot offer their support. Therefore, a patient still can be satisfied with his social relationships, which is reflected by QOL questions of the WHOQOL-Bref such as: ‘How satisfied are you with your social support?’ [23].

An interesting difference was found in our study between the results of the WHOQOL-Bref and the SMFA regarding the physical domain, suggesting that the selection of questionnaires has an influence on the derived conclusions. Patients did not return to their pre-injury level of Physical health on the generic WHOQOL-Bref 6 months post-fracture. The presence of ongoing pain may be disabling in these patients thereby being less satisfied with their physical well-being. However, this is in contrast to the full recovery that patients showed on the SMFA subscale Lower extremity dysfunction. Physical consequences of a LEF may not be limited to the area of the lower extremity, but may influence the physical health of a patient as a whole. These limitations may not be captured by the SMFA items targeting only lower extremity dysfunction.

Our retrospective measurement of the patients’ pre-injury status may produce biases. Watson et al. found that retrospectively measured pre-injury scores of HS and HRQOL were consistently higher compared to population norms [33]. The traumatic event may cause a patient to re-evaluate their pre-injury status with reference to the injured status (i.e., response shift) leading to inflation of pre-injury status. However, the post-injury measurements are assumed to be completed with the same internal standard (i.e., with reference to the injured status). Therefore, the retrospectively collected pre-injury status may be more suitable compared to the application of population norms for comparing post-injury outcomes, although the possibility of a small upward bias should be taken into account [33, 34]. In addition, a limitation of our study is that not all eligible patients for our study, which presented themselves at the Emergency Department during the recruitment period, were actually invited to participate. This study was embedded in hospital daily practice, and patients were sometimes not invited due to busy schedules of the Emergency Department employees. It cannot be excluded that this may produce some bias.

Our study sample was heterogeneous including all types of LEF (i.e., with fractures from toe to hip) using the ICD-10 codes applied at the Emergency Department. Understandably, different fracture types can be followed by various treatment protocols including variation in medicines and rehabilitation. However, we did not found it useful to further classify these types of fractures included in the study because comparing HS, HRQOL, and QOL outcomes of different LEF would involve a larger sample size. Our objective was to describe the course of HS, HRQOL, and QOL of patients with LEF in general, leading to general conclusions of this patient group. We only differentiate between types of treatment in the analyses as a rough measure of injury severity, hypothesizing that patients surgically treated (i.e., higher injury severity) had probably a different course of QOL than nonoperatively treated patients (i.e., lower injury severity). Interaction effects were present for Overall QOL and General health as well as Physical health of the WHOQOL-Bref and Daily life consequences of the SMFA. It is important to note that we did not aim to derive conclusions on which treatment type is superior regarding these patient-reported outcomes because of the different composition of both groups. Significant differences were found on fracture location between treatment groups: All fractures of the toe were treated nonoperatively, while fractures of the upper leg/hip were treated surgically in 96.5 % of the cases. So, a high overlap exists between fracture location and treatment type.

A strength of our study is the use of linear mixed models: We efficiently used the available information of the 171 patients. Another strength is the prospective design of the study; however, our follow-up was limited to 6 months. Study results suggest that recovery still continues 6 months post-fracture [14, 20]. Jurkovich et al. found that overall HS significantly improved twelve months after LEF compared to 6 months post-fracture, but was still worse at 12 months compared to baseline [21]. Therefore, in future research, a longer follow-up of minimal one year is necessary and two years preferable. Regarding clinical practice, individualized occupational training might be offered to patients after LEF to improve instrumental activities of daily living and speed up the recovery of patients’ HS [35]. A further recommendation would be to examine possible predictors of the course of HS, HRQOL, and QOL in patients with LEF: sociodemographic, clinical, and psychosocial variables. In patients with ankle fractures, inconclusive results were found for the predictive value of sociodemographic and clinical variables regarding HS and HRQOL [14]. The possible prognostic value of, for instance, the AO/OTA classification [36] and chronic comorbidities [37] in relation to (HR)QOL should be further explored. In addition, in prior research, social support, illness beliefs, and catastrophic thinking were found to be related to HS and HRQOL in patients after fracture [7, 38, 39]. Finally, although HS and (HR)QOL instruments have in common that they include the physical, psychological, and social domains, the different instruments can encompass different additional domains. The WHOQOL-Bref [23] captures also the domain Environment besides the three core domains of QOL, whereas the SF-36 [16, 40] encompasses eight health domains including separate subscales for Bodily pain and Vitality. Researchers are advised to consider which domains are important in answering their study questions and, subsequently, choose the HS and/or (HR)QOL instruments that capture these different domains fitting their research aims.

In conclusion, patients still report significantly impaired HS, HRQOL, and QOL with regard to several areas of well-being 6 months after LEF compared to their pre-injury status. This is both valid for the patients’ physical health and psychological health 6 months post-fracture. Satisfaction with social relationships is not affected by LEF, indicating that this is not a topic of concern in patients with LEF. In general, HS, HRQOL, and QOL of the surgically treated patient (i.e., higher injury severity) are more affected than in the nonoperatively treated patient (i.e., lower injury severity).
